# A Digital Shade-Matching Device for Dental Color Determination Using the Support Vector Machine Algorithm

**DOI:** 10.3390/s18093051

**Published:** 2018-09-12

**Authors:** Minah Kim, Byungyeon Kim, Byungjun Park, Minsuk Lee, Youngjae Won, Choul-Young Kim, Seungrag Lee

**Affiliations:** 1 Medical Device Development Center, Osong Medical Innovation Foundation, Cheongju, Chungbuk 361-951, Korea; kma2269@gmail.com (M.K.); nick.kimby@gmail.com (B.K.); yachon.park@gmail.com (B.P.); min6434@kbiohealth.kr (M.L.); yjwon000@gmail.com (Y.W.); 2 Department of Electronics Engineering, Chungnam National University, Building E2, 79 Daehangno, Yuseong-gu, Daejeon 305-764, Korea; 3 Department of Electronics Engineering, Kookmin University, 77 Jeongneung-no, Seongbuk-gu, Seoul 02707, Korea

**Keywords:** digital shade-matching device, dental color determination, support vector machine

## Abstract

In this study, we developed a digital shade-matching device for dental color determination using the support vector machine (SVM) algorithm. Shade-matching was performed using shade tabs. For the hardware, the typically used intraoral camera was modified to apply the cross-polarization scheme and block the light from outside, which can lead to shade-matching errors. For reliable experiments, a precise robot arm with ±0.1 mm position repeatability and a specially designed jig to fix the position of the VITA 3D-master (3D) shade tabs were used. For consistent color performance, color calibration was performed with five standard colors having color values as the mean color values of the five shade tabs of the 3D. By using the SVM algorithm, hyperplanes and support vectors for 3D shade tabs were obtained with a database organized using five developed devices. Subsequently, shade matching was performed by measuring 3D shade tabs, as opposed to real teeth, with three additional devices. On average, more than 90% matching accuracy and a less than 1% failure rate were achieved with all devices for 10 measurements. In addition, we compared the classification algorithm with other classification algorithms, such as logistic regression, random forest, and k-nearest neighbors, using the leave-pair-out cross-validation method to verify the classification performance of the SVM algorithm. Our proposed scheme can be an optimum solution for the quantitative measurement of tooth color with high accuracy.

## 1. Introduction

Accurate color evaluation is an important factor in dental restoration procedures [1]. Prosthetic dentistry will only provide satisfactory results if dental restorations are esthetically pleasing. Thus, an accurate determination of tooth color is crucial for obtaining a definitive result. Many of the difficulties that arise in dental aesthetic restorations are related to shade matching [2,3]. In dentistry, achieving a color match depends on a series of visual assessments that are usually communicated between two or more persons, typically the clinician, patient, and technician [2]. When restoring the tooth in a dental clinic, color matching of dental restorative materials with the tooth is performed using a shade guide. The shade guide is a standard model consisting of several shade tabs based on the color distribution of natural teeth. The color of the dental restorative material is based on the shade tabs of the shade guide. Visual color determination with shade guides is the most frequently used shade-matching method in clinical practice [4,5,6]. Because the accuracy of visual color determination has been only 40~60% (also estimated by using shade tabs), there have been many issues in the communication between dentists, patients, and technicians [2,7,8]. To solve this problem, electronic shade-matching devices have become more widespread in the field of dentistry. The most commonly used devices are spectrophotometers and colorimeters. They have been researched and tested in several studies in terms of their accuracy and reliability [9,10,11,12,13,14,15,16,17,18,19,20]. In some studies, colorimeters and spectrophotometers have shown better results than visual color determination in terms of accuracy and reproducibility. However, these devices do not provide image information; therefore, complete information regarding tooth color cannot be obtained. Although a shade-matching device based on a tooth image (SpectroShade, MHT Optic Research AG, Niederhasli, Switzerland) is available, its shade-matching accuracy is low and because of its large head size, it is also uncomfortable to use [21].

In this study, we develop a digital shade-matching device for dental color determination by applying the support vector machine (SVM) algorithm. As a basic hardware platform, an intraoral camera (Whicam story 3, Gooddrs, Incheon, South Korea) with a white light-emitting diode (LED) was used; it can be conveniently used by a patient and a dentist owing to its small head size. To remove the glare area by specular reflection, cross-polarization was performed in front of the LED and camera [22,23].

It is very difficult to obtain images with the same color distribution due to differences in electrical, mechanical, and optical characteristics, even when imaging the same shade tab using several identical cameras. In general, color correction is performed using a color chart [24]. However, since the color chart has a wider range of colors than a tooth, we performed color correction based on the color of the shade guide to better calibrate the tooth color range. The VITA 3D-master (3D; VITA Zahnfabrik, Bad Sackingen, Germany) is one of the most used shade guides and has high performance compared to the classic shade guides, with better organization, a wider range, and a uniform shade distribution [25]. The proposed scheme was evaluated by using eight identical intraoral cameras. Among the developed devices, five devices were used to build a database. Each shade tab of the 3D was evaluated in order and the evaluation was repeated seven times to obtain sufficient information. A total of 35 color data sets for each shade tab were obtained to form the database. By performing measurements using the same 3D shade guide with the other three devices, shade-matching accuracy and reliability could be estimated.

An SVM is a supervised learning model with an associated learning algorithm that analyzes data used for classification analysis [26,27,28]. Because classification among color groups of neighboring shade tabs is important for a shade-matching application, shade-matching by using an SVM algorithm may be an optimum solution for suitable shade tab matching with high accuracy. For comparison, shade matching by using the Euclidean distance between representative colors of shade tabs in the database and the measured color was also performed.

The Commission Internationale de l’Eclairage (CIE) L*a*b* (CIEL*a*b*) color space and color difference formula define color in terms of three coordinate values (L*, a*, b*), which locate the color of an object within a three-dimensional color space [25]. It is used frequently in color research and is based on the color standardization of light sources and observers. In most studies on color evaluation of teeth, the CIE L*a*b* color order system has been used [19,20,29,30]. The CIE L* value is a measure of the lightness of an object, which implies that a perfect black has a CIE L* value of zero and a perfect reflecting diffuser (white) has a CIE L* value of 100. The CIE a* value is a measure of redness (positive value) or greenness (negative value), and the CIE b* value is a measure of yellowness (positive value) or blueness (negative value) [31]. In the CIEL*a*b* color space, the difference between two colors A and B (ΔE) is commonly determined by calculating the Euclidean distance between them. In previous studies, color difference (ΔE) has been used for tooth shade matching [5,32,33,34]. Shade matching using color difference is based on a spherical boundary. However, shade selection error occurs because the color distribution of shade tabs is not spherical. Therefore, we proposed the application of the SVM classification algorithm to shade selection. Using the remaining three devices that did not contribute toward organizing the database, the performance for accuracy and reliability of the proposed scheme was estimated. In addition, the proposed scheme was evaluated through cross validation using eight identical intraoral cameras. The classification algorithm was compared with other classification algorithms, such as logistic regression (LR), random forest (RF), and k-nearest neighbors (kNN) [35,36,37]. Cross validation was performed using the leave-pair-out cross-validation (LPOCV) method [38,39]. LPOCV is a useful method for evaluating the performance of the models of machine learning algorithms, particularly when the number of data is small. In this approach, p datasets are used as validation sets, while the remaining datasets (N-p of the N datasets) are used as training sets. This is repeated for all possible combinations that divide all datasets into training and validation sets. In this study, three device datasets from eight device datasets were assigned as validation sets, and the performance of each algorithm was evaluated.

## 2. Materials and Methods

**Figure 1:**
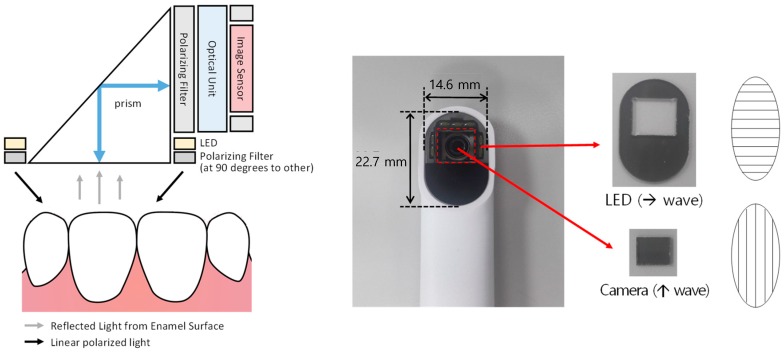
Schematic of the developed digital shade-matching device for dental color determination. LED, light-emitting diode.

The hardware of our proposed digital shade-matching device for dental color determination was realized by applying a cross-polarization scheme to the commercial intraoral camera (Whicam story 3, Gooddrs, Incheon, South Korea) as described in Figure 1. As a light source, a white LED was used and placed around a prism. Two linear polarizers (Edmund Industrial Optics, Barrington, IL, USA) were placed perpendicularly in front of the light source and the prism to enable cross-polarization. A white light source was projected on the tooth surface after it was passed through a horizontal polarizing filter. The reflected or scattered light from the tooth was injected into the prism. Owing to the cross-polarization mechanism, the horizontally polarized reflected light was blocked by the perpendicularly polarizing filter such that color distortion by the specular reflection on the tooth surface was removed. The part of the scattered light from the tooth was reflected by the prism and injected into the image sensor after being passed through an optical unit, such as a lens. The field of view (FOV) of our developed device was 23 × 15 mm^2^. It was sufficient to measure a tooth or shade guide. The size of shade guides of the 3D was ~8.2 × 12 mm^2^. A single frame for evaluation of tooth color was achieved within 200 ms. The frame rate of the device was 5 fps. The software for the proposed device was developed in the Microsoft Visual Studio 2013 (Microsoft, Redmond, WA, USA) environment with Microsoft Foundation Class Library (MFC) and Open Source Computer Vision Library (Open CV).

For color correction, a standard color chart that contained five color squares was designed. The colors of the color squares were determined by the mean color values of the 1M1, 2M2, 3M2, 4M2, and 5M2 models, respectively, of the 3D [40]. The original red, green and blue (RGB) values used to make the standard color chart and RGB values measured by the device were used to obtain a 3 × 3 color correction matrix (CCM), extracted using the least-squares algorithm, for performing color correction [24,41].

**Figure 2:**
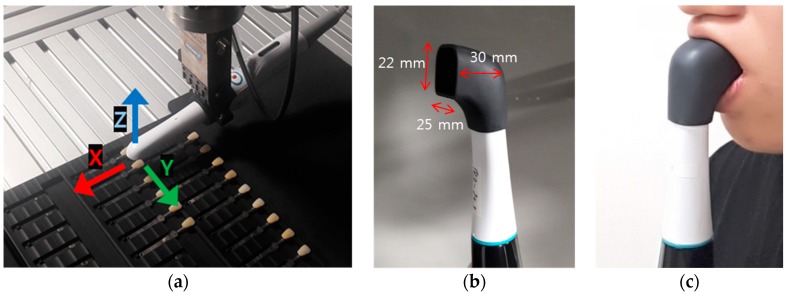
(**a**) Experimental setup for color measurement using the 3D shade guides (**b**) Head cap to block the light from outside (**c**) Clinical application of the developed digital shade-matching device.

To eliminate color errors caused by environmental conditions, a robot arm (LBR iiwa 7 R800, KUKA, Siegen, Germany) with position repeatability of ±0.1 mm was used and a specially designed jig was used to fix the position of the 26 shade tabs of the 3D as shown in Figure 2a. Figure 2b shows a cap equipped on the head of the device to eliminate any color errors caused by the light from outside. Figure 2c shows the clinical application of the developed digital shade-matching device. In this study, color measurement of shade guides was performed on a 632.4 lux LED room light with a color temperature of 5332 K. In total, the size of the device head with the cap was 25 × 22 × 30 mm^3^ so that the back tooth color could also be evaluated.

**Figure 3:**
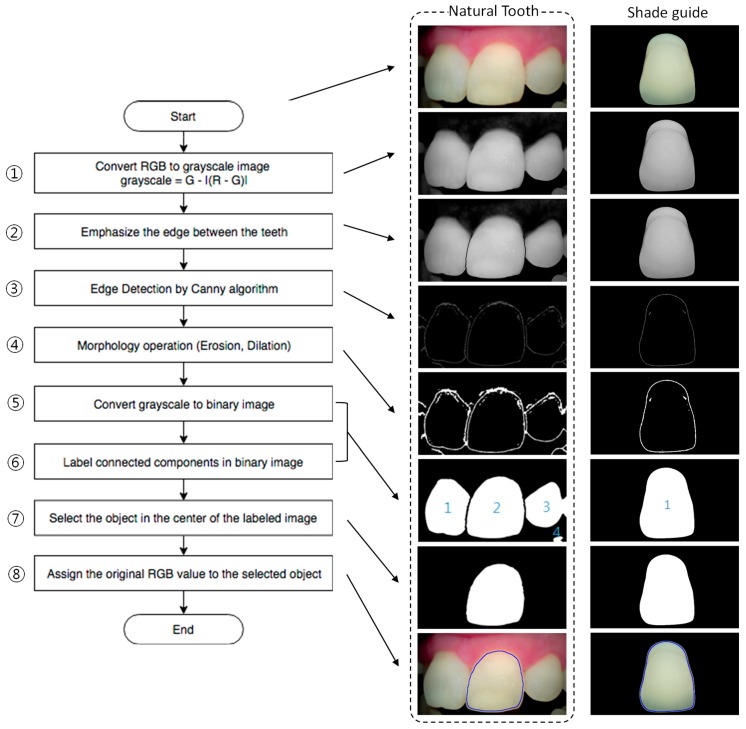
Edge detection procedure.

Figure 3 shows the edge detection procedure to select the shade guide. Although the 3D shade guide was used in this study as a sample to estimate our proposed scheme, the edge detection procedure was considered for real teeth in the clinic. By using the developed device, an image that contains several teeth was obtained as shown in Figure 3. Because the clinic will analyze the patient’s tooth, a process was performed to extract only the central tooth among several teeth. First, the tooth area was differentiated from the gum area by using the formula “grayscale = G − |(R − G)|”. Because the R value of the gum was greater than the R value of the tooth and the G value of the gum was similar to the G value of the tooth, the color difference between the R and G values could be used to convert the image to reveal the tooth area. Then, the edge of each tooth was emphasized by applying a 10 × 20 “Canny mask” and morphology algorithms, such as “erosion” and “dilation.” The grayscale image was converted again to a binary image with a threshold set by the mean value of the grayscale for the edge area. From the binary image, each tooth was distinguished by applying the “labeling” algorithm. The proposed edge detection procedure was also well-applied to emphasize the shade guide from the background. After edge detection of the shade guide, the mean color value was extracted and analyzed.

The evaluation of color, whether visual or through instruments, requires an understanding of the parameters with which color is expressed and measured. In this study, a CIE L*a*b* color order system was used for tooth color evaluation. The conversion formulas from RGB to CIEL*a*b* are as follows:

X = (0.412453∗R + 0.357580∗G + 0.180423∗B)/0.95047;

Y = (0.212671∗R + 0.715160∗G + 0.072169∗B);

Z = (0.019334∗R + 0.119193∗G + 0.950227∗B)/1.08883;

$$\begin{array}{l} \mathrm{if}(X>0.008856)\;\mathrm{then}\;\mathrm{fx}=\mathrm{pow}(X,1/3);\\ \mathrm{else}\;\mathrm{fx}=7.787*X+16/116;\\ \mathrm{if}(Y>0.008856)\;\mathrm{then}\;\mathrm{fy}=\mathrm{pow}(Y,1/3);\\ \mathrm{else}\;\mathrm{fy}=7.787*Y+16/116;\\ \mathrm{if}(Z>0.008856)\;\mathrm{then}\;\mathrm{fz}=\mathrm{pow}(Z,1/3);\\ \mathrm{else}\;\mathrm{fz}=7.787*Z+16/116;\\ L^{*}=116*\mathrm{fy}-16;\\ a^{*}=500*(\mathrm{fx}-\mathrm{fy});\\ b^{*}=200*(\mathrm{fy}-\mathrm{fz}); \end{array}$$

In the CIEL*a*b* color space, the difference between two colors A and B is commonly determined by calculating the Euclidean distance between them [42]. The formula used for calculating color differences is as follows:

ΔE = [(ΔL*)^2^ + (Δa*)^2^ + (Δb*)^2^]^1/2^ (1)

where ΔL*, Δa*, and Δb* are the differences in the color parameters for the two specimens that are being compared.

**Figure 4:**
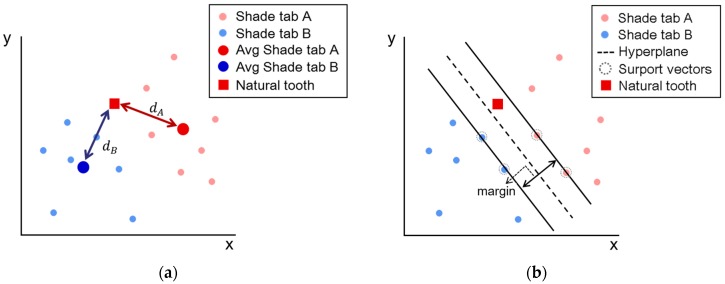
Example for selecting a shade tab by (**a**) the Euclidean distance and (**b**) the support vector machine algorithm. (*x*-axis: Commission Internationale de l’Eclairage (CIE) a* value, *y*-axis: CIE b* value, small red and blue circles: mean colors of shade tabs A and B; large red and blue circles: averaged color of the mean color group; red square: mean color of the natural tooth).

For the shade-matching device, the Euclidean distance between the measured tooth color and the color of the shade tabs in the database can be used to find the suitable shade tab. The shade tab that has the smallest Euclidean distance would be the appropriate one. Figure 4a shows an example for selecting a shade tab by the Euclidean distance. In the a*b* axis of the CIEL*a*b* color space, the filled red circles and filled blue squares represent the measured colors for shade tabs A and B, respectively. Owing to device imperfection, the colors measured by several devices were spread within a certain group in the color space. From the measured colors, the mean color was extracted and this could be the representative color to determine the suitable shade tab by calculating the Euclidean distance using the measured tooth color. In this case, the mean position in the color group and the distance between two neighboring groups may affect the shade matching accuracy because the matched shade tab could be relatively determined by the distance between the measured color and representative colors for the shade tabs.

An SVM is a supervised machine learning algorithm that can be used for classification analysis. In this algorithm, each data item is plotted as a point in n-dimensional space with the value of each feature being the value of a particular coordinate. Then, classification is performed by finding the hyperplane that differentiates the two classes very well. To classify nonlinear data, the classification is performed after mapping to a higher dimension that can be linearly separated by the kernel function. Figure 4b represents an example of selection of a shade tab by an SVM. By using the “CvSVM” function of “Open CV” for the measured data, the support vectors and hyperplane to separate two classes with the maximum margin can be obtained. Here, Gaussian radial basis function (RBF) kernels were used. Based on the color area separated by the hyperplane, a suitable shade tab was determined. In this case, a training data set to find the support vectors and the hyperplane is important because it contains most of the cases that can be measured by different devices or separately measured by the same device. The accuracy and reliability of the developed digital shade-matching device for dental color determination were analyzed by applying the two methods, namely Euclidean distance and the SVM algorithm.

To demonstrate our proposed scheme, eight identical intraoral cameras were developed with the same process of cross-polarization, color calibration, and edge detection. Among these, five devices were used to organize a database for extraction of Euclidean distances, hyperplanes, and support vectors. By using three additional devices that did not contribute toward organizing the database, the performance for accuracy and reliability of our scheme was analyzed. First, the color images for the 26 shade tabs of the 3D were measured in order. After the measurement of the color image for each shade tab, color correction, edge detection, and mean color extraction were sequentially performed. Then, Euclidean distances between the measured tooth color and mean color for all shade tabs in the database were calculated; the shade tab with the smallest Euclidean distance was selected as a matching shade tab. Based on the hyperplanes in the database, a color matching process was performed in addition to the Euclidean distance. Then, the same process was repeated 10 times and the accuracy and reliability of the proposed scheme were analyzed.

**Figure 5:**
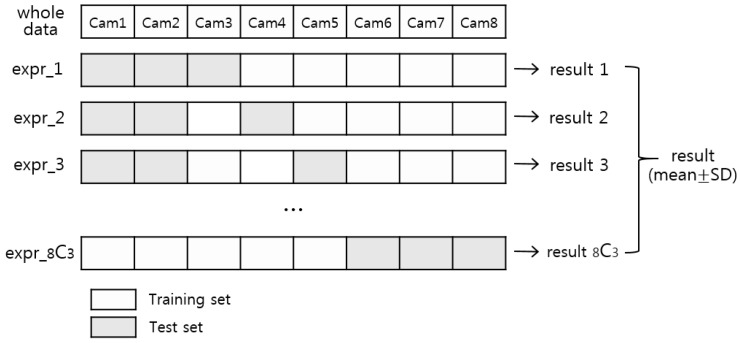
Schematic view of leave-pair-out cross validation.

In addition, the performance of the SVM algorithm and other classification algorithms was evaluated using the LPOCV method. Figure 5 shows the schematic view of the method. In the eight device datasets, three device datasets were assigned as validation sets and the remaining datasets were assigned as training sets. Then, the color matching process was performed based on the classification models in the training sets. This process was repeated for all possible combinations that divided all datasets into training and validation sets, and the accuracy and reliability of the proposed scheme were analyzed.

## 3. Results

**Figure 6:**
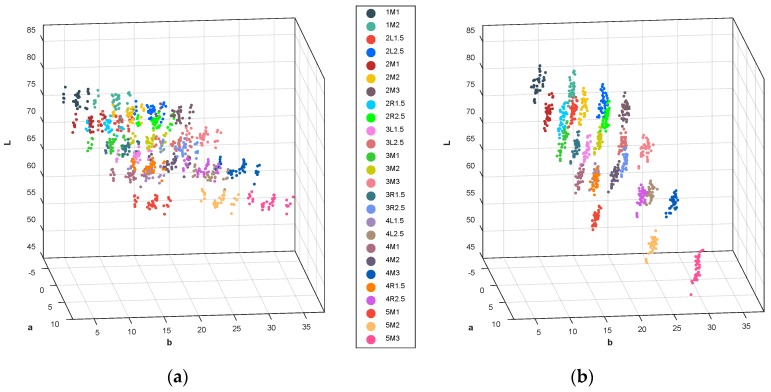
Colors of the Vita 3D master (3D) shade tab measured by the developed digital shade-matching device in the CIEL*a*b* color space (**a**) before color correction and (**b**) after color correction.

To organize a database, 26 shade tabs of the 3D, from 1M1 to 5M3, were measured seven times by using five devices such that a total of 35 color information sets for each shade tab were obtained. Figure 6 shows the mean colors of the 3D measured by the developed digital shade-matching device in the CIEL*a*b* color space. As described in the previous section, color correction was performed by the 3 × 3 CCM after measurement of the tooth image. The effect of color correction is shown in Figure 6a,b (see Supplementary Materials, Videos S1 and S2). The measured color group for each shade tab was more consistently separated from the neighboring group after color correction.

**Figure 7:**
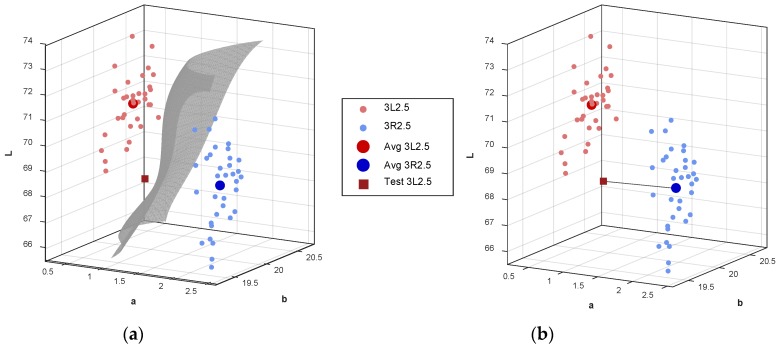

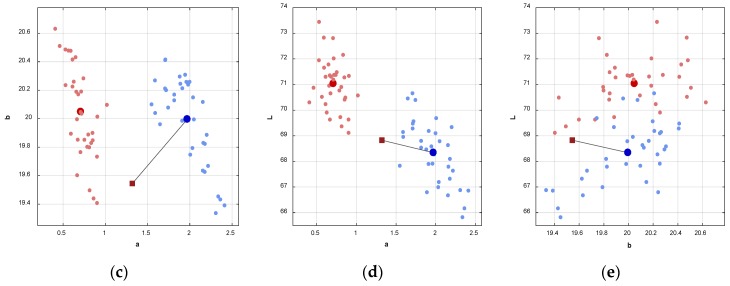
Colors 3L2.5 and 3R2.5 of the 3D shade tab measured by the developed digital shade-matching device in the CIEL*a*b* color space: (**a**) two-dimensional hyperplane; (**b**) Euclidean distance in the three-dimensional color space; (**c**–**e**) Euclidean distance in the two-dimensional color space of the a*b*, L*a*, and L*b* axis. (Small red and blue circles: mean colors of 3L2.5 and 3R2.5 in the database; large red and blue circles: averaged color of the mean color group in the database; red square: mean color of 3L2.5 measured additionally for testing).

Figure 7 shows the measured colors for the two shade tabs 3L2.5 and 3R2.5. The small red and blue circles represent the 35 measurements of the mean colors of 3L2.5 and 3R2.5, respectively (see Supplementary Materials, Video S3). The large red and blue circles denote the averaged color of their mean color group. The red square is the mean color of 3L2.5 measured by an additional device that was not used for organizing the database. From the measured colors, a two-dimensional hyperplane for SVM could be obtained as shown in Figure 7a. Based on the hyperplane, the colors on the left side were corrected to 3L2.5 such that the measured test color could be well-determined to be 3L2.5. However, the Euclidean distance between the measured test color and the averaged color of 3R2.5 was smaller than the Euclidean distance between the measured test color and the averaged color value of 3L2.5 as shown in Figure 7b–e. In this case, the measured test color was decided to be 3R2.5 by the Euclidean distance.

Table 1 shows the experimental results to evaluate the accuracy of the developed digital shade-matching device with the Euclidean distance and the SVM algorithm. The accuracy could be evaluated by the matching rate. For all devices, the matching rate for the SVM algorithm was more stable compared to the Euclidean distance. In the case of the Euclidean distance, the matching rate for the first case of device #3 was very poor at approximately 50%. This means that 13 shade tabs were not matched. However, the matching rate could be improved to 84.6% when the SVM algorithm was applied. For all cases except 3, the matching rate for the SVM algorithm was higher than the matching rate for the Euclidean distance. On average, a matching rate of more than 90% was achieved for the SVM algorithm. The standard deviation of the matching rate for the SVM algorithm was also better than that of the Euclidean distance for 10 cases.

The developed digital shade matching device would be considered 100% reliable for measuring a 1M1 shade tab if all 10 measurements were the same, even if the measurements did not correspond to 1M1. Overall reliability for the device to be analyzed was then calculated as the average reliability across all 26 shade tabs. For the Euclidean distance, the reliability was 88.8(0.86), 80.8(1.00), and 75.41(1.43) for devices #1, #2, and #3, respectively, where the number outside the bracket is the average reliability and the number inside the bracket is the standard deviation (failure rate). The reliability for the SVM algorithm was 95.0(0.36), 91.9(0.71), and 93.5(0.76) for devices #1, #2, and #3, respectively.

Table 2 shows the performance of the suggested scheme obtained using LPOCV. For comparison, the table also shows the performance of other classification algorithms, such as LR, RF, and KNN. The reliabilities of the RF, KNN, and SVM algorithms are 89.3, 96.1, and 96.9, respectively.

## 4. Discussion and Conclusions

We developed a digital shade-matching device for dental color determination by applying the SVM algorithm. In restorative dentistry, a dentist commonly encounters the challenge of replicating the color of natural teeth. The color of the dental restorative material is based on shade guides. Therefore, it is important to select the shade tab that most closely resembles the patient’s teeth. The most popular and traditional method of shade selection in dentistry is through the use of visual selection with a prefabricated shade guide. However, color duplication using this process is unreliable and inconsistent. Chairside color matching with shade guides is considered subjective and difficult because of variable viewer interpretation and environmental influences, such as fatigue of the human eye, aging, emotions, lighting conditions, level of experience, and physiological variables, such as color blindness. To solve this problem, electronic shade matching devices, such as spectrophotometers and colorimeters, have been used in dentistry. However, these devices do not provide image information owing to their point measurement scheme. Although a shade-matching device based on a tooth image is available, its shade-matching accuracy is low and because of its large head size, it is also uncomfortable to use.

In this study, the typically used intraoral camera was used as a hardware platform to enhance usability because of its small head size and light weight. By applying the cross-polarization scheme, the ratio between the color distortion area caused by specular reflection and the whole tooth area was reduced from 11.24% to 0.28%. Because the color performance of the manufactured LEDs and cameras was different from each other owing to hardware imperfection, color calibration was required after measuring the color image. Because the tooth color area was a small portion of the RGB color space, color calibration with colors of several representative shade tabs was desirable for accurate digital shade matching while pure RGB colors were used for color calibration for commonly used display devices, such as monitors, endoscopes, and mobile phones.

In previous studies, shade tabs have been used as a standard sample to demonstrate their dental shade-matching scheme [7,21,43,44]. In other studies, the visual evaluation of real teeth is performed by measuring color differences, but these measurements do not assess the matching rate [9,10,11,12,13,14,15,16,17,18,19,20]. Thus, they are not suitable for evaluating the matching rate for real teeth. Therefore, we used the 3D as a test sample.

Eight devices were developed and implemented for this study. Five of them were used to set a database and the remaining devices were used to evaluate the performance of our suggested scheme. Each shade tab was measured by five devices in order. Then, the measurement was repeated seven times such that 35 color information sets for a single shade tab were obtained to set the database. From the database, the hyperplanes and support vectors for 26 3D shade guides were extracted and used in the SVM algorithm. For comparison, the mean color values for 26 3D shade guides were also acquired and used to obtain the Euclidean distance. In the CIEL*a*b* color space, the difference between two colors A and B is commonly determined by calculating the Euclidean distance between them. For a shade-matching application, the Euclidean distance between the measured tooth color and mean colors of all shade tabs in the database could be used to find a suitable shade tab. However, shade matching with Euclidean distance is very sensitive. For a single device (device #3 in Table 1), the matching rate varied from 50% to 96.2%. The color group for each shade guide was not symmetric with the mean color as the center and Euclidean distance for some measured color was not the minimum with the color of the corresponding shade tab. This mainly resulted in shade-matching errors. The matching rate could be enhanced by applying the SVM algorithm. In this case, the measured color inside a boundary (hyperplane) of the color group of some shade guide was matched to that shade guide such that the shade-matching condition was more stable from 84.6% to 100.0%. Moreover, the shade-matching accuracy and reliability were also better than those obtained using the Euclidean distance method. The SVM algorithm was more suitable than the color difference method for classifying nonlinear shade distributions and performing shade matching.

Cross validation was performed; this is a method of estimating the prediction and classification performance of a model for new data [38,39]. The cross-validation results of the SVM algorithm were compared with those of other classification algorithms, such as LR, RF, and KNN. The matching rate of LR and RF was lower than that of SVM. KNN had similar matching results with SVM; however, its standard deviation was higher. Therefore, SVM showed more stable performance. In this study, only five devices were used to obtain the hyperplanes and support vectors. If machine learning is performed using more devices, the shade-matching accuracy and reliability can also be enhanced. The hyperplane obtained by the SVM may also be used as a guide to evaluate whether the product is well-manufactured or not.

Experimental results show that the proposed digital shade-matching device for dental color determination using the SVM algorithm may be an optimum solution for quantitative measurement of tooth color. In previous studies on other shade-matching devices for dental color determination, the matching rates of spectroshade, shadevision, vita easyshade, and shadescan were 80.2%, 84.8%, 92.6%, and 66.8%, respectively [21]. Here, the 3D shade guide was used as a sample. Researchers have also tried to enhance the matching rate by using spectrophotometric and computer matching techniques for spectrophotometers [9,45]. However, the matching rates achieved with their methods were only 83.3% for the spectrophotometric method and 61.1% for the computer matching method. Digital analysis of the dental image (experimental software-DetColorDent 1.1, Babes-Bolyai University, Cluj-Napoca, Romania) was applied to assess the color parameters of the tooth. However, it needs further improvements to the accuracy of CIE L*a*b* values [20].

The measured color varies according to the measurement position, which was fixed in this study for a reliable experiment. Therefore, in future work, the optimum range of the position and angle between the device head and tooth surface would be analyzed and methods to determine the optimum position of the device would also be suggested.

## Figures and Tables

**Table 1:** Accuracy of the developed digital shade-matching device using the Euclidean distance and the support vector machine (SVM) algorithm.

Method	Device	Matching Rate (%, 100 × (# of Matching Shade Tabs/26))										Average	SD
		1	2	3	4	5	6	7	8	9	10		
ΔE (Euclidean distance)	#1	84.6	96.2	100.0	80.8	88.5	88.5	80.8	88.5	88.5	84.6	88.1	6.1
	#2	96.2	96.2	57.7	76.9	76.9	84.6	92.3	57.7	76.9	57.7	77.3	15.4
	#3	50.0	61.5	61.5	53.8	96.2	76.9	80.8	80.8	92.3	92.3	74.6	16.8
SVM	#1	96.2	92.3	100.0	88.5	88.5	100.0	100.0	96.2	96.2	92.3	95.0	4.46
	#2	92.3	88.5	96.2	96.2	96.2	84.6	92.3	84.6	92.3	96.2	91.9	4.6
	#3	84.6	96.2	92.3	92.3	100.0	92.3	88.5	88.5	100.0	100.0	93.5	5.45

**Table 2:** Accuracy of the developed digital shade-matching device using the SVM algorithm and other classification algorithms by leave-pair-out cross validation (*p* = 3).

Classification Method	Matching Accuracy (%)	SD
Logistic Regression	77.2	16.58
Random Forest	89.3	3.93
K-Nearest Neighbors	96.1	1.47
Support Vector Machine	96.9	1.37

## Supplementary Materials

The following are available online at http://www.mdpi.com/1424-8220/18/9/3051/s1, Video S1: Colors of the 3D Shade tab measured by the developed digital shade-matching device in the CIEL*a*b* color space before color correction, Video S2: Colors of the 3D Shade tab measured by the developed digital shade-matching device in the CIEL*a*b* color space after color correction, Video S3: Colors of the 3D tabs 3L2.5 and 3R2.5 measured by the developed digital shade-matching device in the CIEL*a*b* color space.
