# Supplementary figures and images for: A Digital Shade-Matching Device for Dental Color Determination Using the Support Vector Machine Algorithm
Source: Sensors (Basel). 2018 Sep 12;18(9):3051. doi: 10.3390/s18093051 (PMC6165317; doi:10.3390/s18093051)

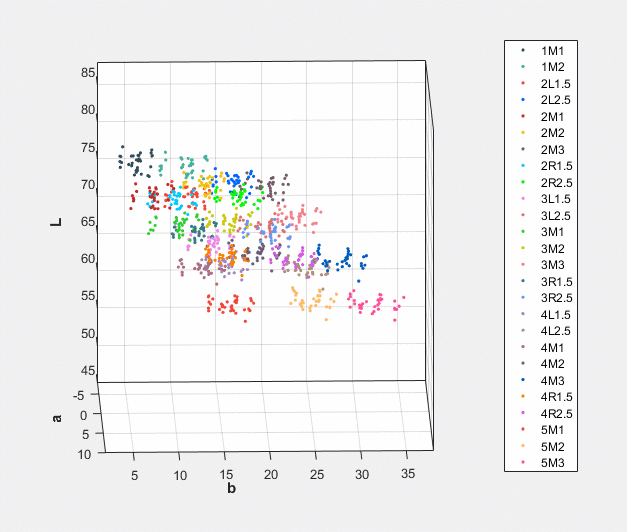

Supplement: Supplementary file 1 [file sensors-18-03051-s001.zip › VideoS1.gif]

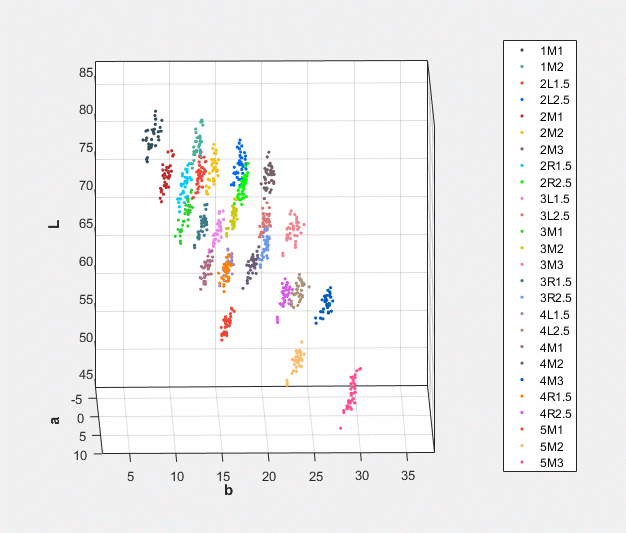

Supplement: Supplementary file 1 [file sensors-18-03051-s001.zip › VideoS2.gif]

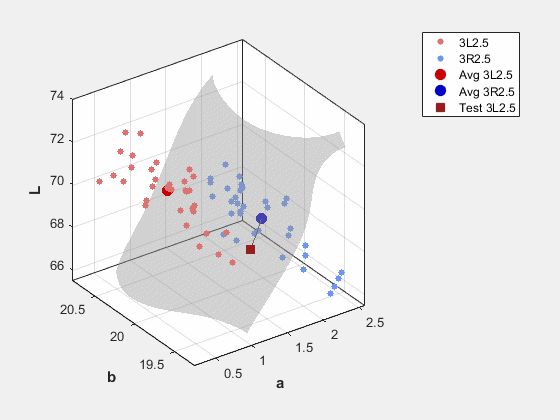

Supplement: Supplementary file 1 [file sensors-18-03051-s001.zip › VideoS3.gif]
